# Prevalence and Risk Factors for Financial Toxicity Among Sarcoma Patients During and Following Treatment Across the Lifespan

**DOI:** 10.1002/cnr2.70686

**Published:** 2026-09-30

**Authors:** Michael J. Robinson, Lili Sun, Tatsuki Koyama, Emma A. Schremp, Grace Aronoff, Tulio Guimaraes, Taylor A. Stokes, Elizabeth J. Davis, Vicki Keedy, Scott C. Borinstein, Herbert S. Schwartz, Jennifer L. Halpern, Joshua M. Lawrenz, Daniel Johnson, Timothy Su, Leo Luo, Travis Osterman, Tuya Pal, Ben H. Park, Xiao‐Ou Shu, Debra L. Friedman, Eric T. Shinohara

**Affiliations:** ^1^ Division of Pediatric Hematology and Oncology, Department of Pediatrics Vanderbilt University Medical Center Nashville Tennessee USA; ^2^ Department of Biostatistics Vanderbilt University Medical Center Nashville Tennessee USA; ^3^ Division of Hematology and Oncology, Department of Medicine Vanderbilt University Medical Center Nashville Tennessee USA; ^4^ Department of Orthopedic Surgery and Rehabilitation Vanderbilt University Medical Center Nashville Tennessee USA; ^5^ Division of Epidemiology, Department of Medicine Vanderbilt University Medical Center Nashville Tennessee USA; ^6^ Department of Radiation Oncology Vanderbilt University Medical Center Nashville Tennessee USA; ^7^ Department of Biomedical Informatics Vanderbilt University Medical Center Nashville Tennessee USA; ^8^ Division of Genetic Medicine, Department of Medicine Vanderbilt University Medical Center Nashville Tennessee USA

**Keywords:** COST, financial toxicity, PROMIS‐57, sarcoma

## Abstract

**Background:**

Financial toxicity (FT) describes the impact of cancer and treatment‐related financial burden. We evaluated the prevalence and risk factors for FT among sarcoma patients.

**Methods:**

A sarcoma cohort was constructed to assess health outcomes. FT was measured via “COmprehensive Score for financial Toxicity” (COST), where lower scores indicate greater FT. COST scores were evaluated in association with patient demographics, clinical data, and psychosocial/functional domains (PROMIS‐57) using multivariable linear regression. Subgroup analyses evaluated patients on‐ versus off‐therapy.

**Results:**

Among 1125 participants (515 on/610 off therapy), median COST was 27.0 (IQR 18.0, 35.0), with absent, mild, moderate, and severe FT seen in 55.4%, 29.2%, 15.1%, and 0.4%, respectively. Lower FT was associated with increasing age (+1.28; 95% CI 0.86, 1.70), male sex (+1.93; 0.78, 3.08), and higher income (+0.87; 0.72, 1.01). Increased FT was associated with non‐White race (−1.79; −3.41, −0.13), larger households (−0.73; −1.25, −0.22), metastatic disease (−2.36; −3.80, −0.91), and active treatment (−1.61; −2.97, −0.24). Subgroup analyses demonstrated similar results regarding age, sex, income, and metastatic disease among on‐therapy and age, income, and household members among off‐therapy. Eight hundred and thirty‐two participants completed both the PROMIS‐57 and COST. FT was associated with anxiety (−0.77; −1.45, −0.08), depression (−2.11; −2.96, −1.26), pain interference (−1.36; −2.17, −0.55), and sleep disturbance (−0.68; −1.35, −0.004).

**Conclusion:**

Almost half of participants experienced FT. Risk factors included non‐White race, active therapy, larger households, and metastatic disease. Anxiety, depression, pain interference, and sleep disturbance were associated with FT. Screening for FT is needed to develop targeted interventions.

## 1 Introduction

Sarcomas represent a diverse group of rare solid tumors [1, 2], for which survival has increased over the last several decades [3, 4, 5, 6, 7, 8, 9, 10, 11]. Simultaneously, the financial cost of cancer treatment has also increased [12, 13, 14]. With an increasing population of sarcoma survivors experiencing greater financial burden, there is a growing need to understand factors associated with financial toxicity (FT) in order to develop interventions to assist patients and families.

FT is not financial loss, but rather describes the financial distress and burden experienced by patients. Studies have demonstrated a negative impact on quality of life (QoL), reduced treatment adherence, missed follow‐up care, and decreased survival associated with increased financial burden among cancer patients [15, 16, 17, 18, 19, 20, 21, 22, 23]. However, much of this data have been reported among more common cancers, such as leukemia, prostate, breast, colorectal, and lung [14, 17, 20, 22, 24, 25, 26].

Limited studies of FT among sarcoma patients of any age include European cohorts [27, 28] or focus on patients receiving palliative treatments [29]. Yet, FT has been shown to be a clinically relevant and unmet need among this group [30]. In countries with universal healthcare, greater financial distress was seen among patients with sarcoma compared to an age and gender‐matched general population [27].

Sarcoma therapy and its adverse side effects may place patients at particularly increased risk for FT and hardship, compared to the general population and most other forms of malignancies [27, 31]. Treatment frequently includes chemotherapy, surgery, radiation, or targeted therapy, resulting in physical and mobility impairment. Repeated clinical encounters in turn may limit earning potential while increasing expenses [27, 30].

Herein, we present data on the prevalence of and risk factors for FT among a cohort of adult sarcoma patients.

## 2 Methods

### 2.1 Study Population and Data Collection

A cohort of contemporarily treated sarcoma patients, “Cohort to Augment the Understanding of Sarcoma Survivorship Across the Lifespan” (CAUSAL), was constructed from the Vanderbilt University Medical Center (VUMC) Sarcoma Center to evaluate oncologic and nononcologic outcomes and their determinants. The study was approved by the VUMC Institutional Review Board and informed consent was obtained from all participants. Participants eligible for participation in CAUSAL are identified using an informatics‐driven approach within the electronic health record (EHR), based on prespecified inclusion criteria. Inclusion criteria for CAUSAL include patients of any age followed at VUMC with a confirmed history of sarcoma, with or without evidence of persistent disease, who are English‐speaking. No compensation is provided to enrollees. Participants included in the present analysis were age 18 or older at the time of CAUSAL enrollment.

At study enrollment, surveys were conducted through Research Electronic Data Capture (REDCap) to collect information on patient demographic characteristics, lifestyle factors, and outcome measures (including financial toxicity and psychosocial and functional outcomes). Surveys and questionnaires conducted via REDCap were administered on electronic tablets at enrollment. For participants who expressed difficulties using electronic devices, paper questionnaires were made available.

Insurance, clinical, and sarcoma treatment data were abstracted from the electronic health records via an informatics approach and manual chart review when appropriate to enhance data quality and accuracy.

### 2.2 FT and Patient Reported Outcome Measures

Self‐reported FT was assessed via the “COmprehensive Score for financial Toxicity” (COST), a validated measure of FT among individuals with cancer [16, 32]. COST consists of 11 items, with each item scored on a five‐point Likert scale, with responses of Not At All (0), A Little Bit (1), Some‐What (2), Quite A Bit (3), and Very Much (4). Participants are asked to select a response based on their experience over the prior 7 days. Responses to negatively worded questions are reversed scored. At completion, the sum of all items is obtained and multiplied by the number of completed items in the scale and then divided by the number of items answered. This results in the final score (range 0–44), where lower scores indicate greater FT. COST scores were stratified as follows: 0 = severe FT, 1–13 = moderate FT, 14–25 = mild FT, and ≥ 26 = no FT. Although no single consensus stratification system exists, with cut offs varying across studies, the thresholds applied here were proposed by de Souza et al. and have been adopted in subsequent published works [32, 33, 34, 35].

Patient‐reported psychosocial and functional outcomes were assessed via the Patient‐Reported Outcomes Measurement Information System (PROMIS)‐57 Profile (version 2.1) [36], that measures the following domains: Anxiety, Depression, Fatigue, Pain Interference, Physical Function, Sleep Disturbance, and Ability to Participate in Social Roles and Activities. Each domain comprises eight items rated on a five‐point Likert scale (Never = 1, Rarely = 2, Sometimes = 3, Often = 4, and Always = 5). Domain scores were calculated according to the PROMIS‐57 scoring manual and converted into a standardized T‐score with a population mean of 50 and a standard deviation (SD) of 10. Higher T‐scores indicate greater levels of the measured construct.

### 2.3 Statistical Analyses

Patient characteristics were described with medians and quartiles for continuous variables, and frequencies for categorical variables. Multivariable linear regression was applied to analyze continuous outcomes (COST score). Covariates included in the models were demographic variables (age at enrollment, sex, race, ethnicity, education level, yearly household income, insurance, and household members supported by the household income), disease and treatment variables (recurrence, treatment status, on versus off therapy, off‐treatment duration, metastatic disease, and tumor site). Covariates of interest were selected due to their prior evaluation in the literature, with most demonstrating a relationship with financial toxicity, hardship, or burden among sarcoma or mixed‐cancer cohorts in the literature [18, 23, 27, 37, 38, 39, 40, 41, 42]. A subgroup analysis was conducted by stratifying the cohort into on‐therapy and off‐therapy groups. In these analyses, the same covariates were included in the models excluding treatment‐related variables. Among participants who completed all PROMIS‐57 domains, additional multivariable linear regression models were performed that included the above covariates as well as all PROMIS‐57 domain T‐scores (anxiety, depression, fatigue, pain, physical function, sleep disturbance, and social roles).

Multicollinearity was assessed using the variance inflation factor (VIF). Values greater than 5 are generally considered problematic; in our models, all VIF values were below 5, indicating no major multicollinearity concern. All statistical analyses were performed using R Statistical Software (version 4.3.1; R Foundation for Statistical Computing, Vienna, Austria). A two‐sided *p*‐value < 0.05 was considered statistically significant.

Missing data were handled by defining the main analytic cohort as participants with a nonmissing COST score, the primary outcome. Missing covariate data in the multivariable regression were addressed using multiple imputation. Analyses involving PROMIS domains were limited to participants with complete COST and PROMIS data.

## 3 Results

### 3.1 Study Population

A total of 1499 adult sarcoma patients (age ≥ 18 years) treated at VUMC were enrolled in the study between April 2022 and August 2025 and 1125 completed the COST measure. Among them, at enrollment 515 were in active therapy and 610 were off therapy. The median age of the study participants at the time of the COST assessment was 56.3 years (IQR 41.5, 67.3). 54.7% of participants were female, 85.2% identified as White, 69.7% were married, 48.1% were employed, 35.2% were retired, 64.8% obtained an education at or above college level and 51.9% described a household income > $70 000. Commercial health insurance was held by 45.9% of participants, while 43.0% had government sponsored insurance. Regarding disease characteristics, 76.4% had localized disease, 20.2% had relapsed/recurrent disease, and 48.8% had their primary disease located within an extremity (Table 1).

**TABLE 1:** Participant demographic and disease characteristics among adult participants who completed COST.

Characteristics	Off therapy (*n* = 610)		On therapy (*n* = 515)		Total (*N* = 1125)	
Median age (IQR)	56.4 (40.9, 67.0)		56.2 (42.2, 67.7)		56.3 (41.5, 67.3)	
Gender						
Male	249	40.8%	261	50.7%	510	45.3%
Female	361	59.2%	254	49.3%	615	54.7%
Race						
White	535	87.7%	423	82.1%	958	85.2%
Other	75	12.3%	92	17.9%	167	14.8%
Missing/prefer not to answer	0	0.0%	0	0.0%	0	0.0%
Household income						
< $25 000	56	9.2%	47	9.1%	103	9.2%
$25 000–$45 00	78	12.8%	81	15.7%	159	14.1%
$45 000–$65 000	88	14.4%	65	12.6%	153	13.6%
$65 000–$70 000	35	5.7%	27	5.2%	62	5.5%
$70 000–$90 000	68	11.1%	59	11.5%	127	11.3%
$90 000–$120, 000	94	15.4%	73	14.2%	167	14.8%
$120 00–$150 000	59	9.7%	57	11.1%	116	10.3%
> $150 000	107	17.5%	67	13.0%	174	15.5%
Missing	25	4.1%	39	7.6%	64	5.7%
Household members						
0	0	0.0%	1	0.2%	1	0.1%
1	133	21.8%	103	20.0%	236	21.0%
2	289	47.4%	248	48.2%	537	47.7%
3	0	0.0%	64	12.4%	144	12.8%
4	75	12.3%	55	10.7%	130	11.6%
5	19	3.1%	25	4.9%	44	3.9%
6	4	0.7%	12	2.3%	16	1.4%
7	5	0.8%	2	0.4%	7	0.6%
8	2	0.3%	0	0.0%	2	0.2%
Missing	83	13.6%	5	1.0%	98	8.7%
Disease extent						
Localized	528	86.6%	332	64.5%	860	76.4%
Metastatic	81	13.3%	182	35.3%	263	23.4%
Missing	1	0.2%	1	0.2%	2	0.2%
Relapse/recurrent disease						
No	485	79.5%	408	79.2%	893	79.4%
Yes	122	20.0%	105	20.4%	227	20.2%
Missing	3	0.5%	2	0.4%	5	0.4%
Site of primary disease						
Trunk	148	24.3%	184	35.7%	332	29.5%
Extremity	354	58.0%	195	37.9%	549	48.8%
Head and neck	44	7.2%	41	8.0%	85	7.6%
Retroperitoneal	62	10.2%	94	18.3%	156	13.9%
Missing	2	0.3%	1	0.2%	3	0.3%
Common diagnoses (min. 10)						
Ewing sarcoma	32	5.2%	13	2.5%	45	4.0%
Desmoid	39	6.4%	52	10.1%	91	8.1%
Gastrointestinal stromal tumor (GIST)	37	6.1%	70	13.6%	107	9.5%
Osteosarcoma	40	6.6%	21	4.1%	61	5.4%
Chondrosarcoma	59	9.7%	13	2.5%	72	6.4%
Giant Cell Tumor	24	3.9%	4	0.8%	28	2.5%
Synovial Sarcoma	22	3.6%	11	2.1%	33	2.9%
Dedifferentiated liposarcoma	15	2.5%	22	4.3%	37	3.3%
Tenosynovial giant cell tumor (TGCT)	17	2.8%	12	2.3%	29	2.6%
Leiomyosarcoma	38	6.2%	62	12.0%	100	8.9%
Myxofibrosarcoma	34	5.6%	29	5.6%	63	5.6%
Myxoid liposarcoma	15	2.5%	11	2.1%	26	2.3%
Pleomorphic sarcoma	16	2.6%	11	2.1%	27	2.4%
Undifferentiated pleomorphic sarcoma	39	6.4%	32	6.2%	71	6.3%
Well‐differentiated liposarcoma	48	7.9%	7	1.4%	55	4.9%
Other	135	22.1%	145	28.2%	280	24.9%
Marital status						
Married	427	70.0%	357	69.3%	784	69.7%
Divorced	61	10.0%	46	8.9%	107	9.5%
Separated/widowed	38	6.2%	37	7.2%	75	6.7%
Never married	79	13.0%	70	13.6%	149	13.2%
Missing	5	0.8%	5	1.0%	10	0.9%
Employment status						
Employed	311	51.0%	230	44.7%	541	48.1%
Unemployed	53	8.7%	83	16.1%	136	12.1%
Retired	218	35.7%	178	34.6%	396	35.2%
Missing	28	4.6%	24	4.7%	52	4.6%
Highest level of education						
College or higher	422	69.2%	307	59.6%	729	64.8%
High school or less	183	30.0%	200	38.8%	383	34.0%
Missing	5	0.8%	8	1.6%	13	1.2%
Insurance						
Commercial	286	46.9%	230	44.7%	516	45.9%
Government	250	41.0%	234	45.4%	484	43.0%
Self‐pay	64	10.5%	33	6.4%	97	8.6%
Missing	10	1.6%	18	3.5%	28	2.5%

### 3.2 COST Scores

Median COST score for all participants was 27.0 (IQR 18.0, 35.0). 55.4% had a COST score ≥ 26 (consistent with no FT), 29.2% of scores ranged between 14 and 25, 15.1% were between 1–13, and 0.4% scored 0, corresponding to mild, moderate, and severe FT, respectively. Among the 515 participants on therapy, 51.1% of scores were ≥ 26, 29.9% between 14 and 25, 18.6% between 1 and 13, and 0.4% scored 0. Among the 610 participants off therapy, 59.0% of scores were ≥ 26, 28.5% between 14 and 25, 12.1% between 1 and 13, and 0.3% scored 0. (Table 2).

**TABLE 2:** Preliminary COST results and stratification for adult participants (Age 18 and older).

Cost stratification^a^	Off therapy (*n* = 610)		On therapy (*n* = 515)		Total (*N* = 1125)	
No financial toxicity	360	59.0%	263	51.1%	623	55.4%
Mild financial toxicity	174	28.5%	154	29.9%	328	29.2%
Moderate financial toxicity	74	12.1%	96	18.6%	170	15.1%
Severe financial toxicity	2	0.3%	2	0.4%	4	0.4%

^a^ Stratification system: Score of 0 = Severe, 1–13 = Moderate, 14–25 = Mild, and ≥ 26 = No toxicity.

### 3.3 Demographic and Disease Related Risk Factors for Financial Toxicity

Multivariable regression for COST scores among all participants demonstrated that lower FT was associated with increasing participant age (per 10‐year increase) by 1.28 points (95% CI 0.86, 1.70; *p* = < 0.001), male gender by 1.93 points (0.78, 3.08; *p* = 0.001) and rising household income (per $10 000 increase) by 0.87 points (0.72, 1.01; *p* = < 0.001). Conversely, higher FT was associated with non‐White race by −1.77 points (−3.41, −0.13; *p* = 0.035), increasing household members (per 1 person increase) by −0.73 points (−1.25, −0.22; *p* = 0.0055), metastatic disease by −2.36 points (−3.80, −0.91; *p* = 0.0015), and active treatment by −1.61 points (−2.97, −0.24; *p* = 0.021) (Table 3).

**TABLE 3:** Multivariate linear regression model for COST scores among all adult participants (*N* = 1125).

Variable	Effect	95% CI	*p*
Age at enrollment (per 10‐year increase)	1.28	(0.86, 1.70)	< 0.001
Gender (male vs. female)	1.93	(0.78, 3.08)	0.001
Race (Non‐White vs. White)	−1.77	(−3.41, −0.13)	0.035
Ethnicity (Hispanic/Latino vs. Non‐Hispanic/Non‐Latino)	0.26	(−2.79, 3.32)	0.87
Income (per 10 k increase)	0.87	(0.72, 1.01)	< 0.001
Education level (high school or less vs. college or higher)	0.09	(−1.17, 1.34)	0.89
Household members (per 1 person increase)	−0.73	(−1.25, −0.22)	0.0055
Insurance			
Government vs. commercial	1.32	(−0.16, 2.79)	0.08
Self‐pay vs. commercial	−0.45	(−2.63, 1.73)	0.68
Treatment (On vs. Off)	−1.61	(−2.97, −0.24)	0.021
Off treatment duration (per 12‐month increase)	−0.02	(−0.20, 0.16)	0.83
Site of primary disease			
Extremity vs. trunk	0.33	(−1.02, 1.69)	0.63
Head and neck vs. trunk	0.59	(−1.72, 2.91)	0.61
Retroperitoneal vs. trunk	−0.39	(−2.22, 1.45)	0.68
Disease extent (metastatic vs. localized)	−2.36	(−3.80, −0.91)	0.0015
Recurrence/relapse (Yes vs. No)	−0.10	(−1.55, 1.35)	0.89

Among the 610 off therapy participants who completed the COST measure, multivariable regression demonstrated that lower FT was associated with increasing age at enrollment (per 10‐year increase) by 1.37 points (0.83, 1.92; *p* = < 0.001) and household income (per $10 000 increase) by 0.94 points (0.76, 1.12; *p* = < 0.001). Increasing household members (per 1 person increase) showed a negative association with COST by −0.79 points (−1.47, −0.11; *p* = 0.023), indicative of higher FT (Table 4).

**TABLE 4:** Multivariate linear regression model for COST scores among adult participants off therapy (*n* = 610).

Variable	Effect	95% CI	*p*
Age at enrollment (per 10‐year increase)	1.37	(0.83, 1.92)	< 0.001
Gender (male vs. female)	1.24	(−0.29, 2.77)	0.11
Race (Non‐White vs. White)	−1.10	(−4.30, 0.31)	0.09
Ethnicity (Hispanic/Latino vs. Non‐Hispanic/Non‐Latino)	−0.74	(−4.67, 3.19)	0.71
Income (per 10 k increase)	0.94	(0.76, 1.12)	< 0.001
Education level (high school or less vs. college or higher)	0.22	(−1.43, 1.87)	0.8
Household members (per 1 person increase)	−0.79	(−1.47, −0.11)	0.023
Insurance			
Government vs. commercial	1.35	(−0.60, 3.30)	0.17
Self‐pay vs. commercial	0.62	(−2.00, 3.24)	0.64
Duration of time off treatment (per 12‐month increase)	−0.04	(−0.24, 0.15)	0.68
Site of primary disease			
Extremity vs. trunk	0.78	(−1.03, 2.59)	0.4
Head and neck vs. trunk	1.64	(−1.46, 4.75)	0.3
Retroperitoneal vs. trunk	−0.39	(−3.14, 2.36)	0.78
Disease extent (metastatic vs. localized)	−1.94	(−4.31, 0.43)	0.11
Recurrence/relapse (Yes vs. No)	0.18	(−1.79, 2.15)	0.86

Among the 515 on therapy participants who completed the COST measure, lower FT was associated with increasing age at enrollment (per 10‐year increase) by 1.11 points (0.47, 1.76; *p* = < 0.001), increasing household income (per $10 000 increase) by 0.83 points (0.58, 1.08; *p* = < 0.001), and male gender by 2.67 points (0.90, 4.43; *p* = 0.0032). Metastatic disease was associated with a lower COST score by −2.89 (−4.85, −0.92; *p* = 0.0041) points, indicating higher FT (Table 5).

**TABLE 5:** Multivariate linear regression model for COST scores among adult participants on therapy (*n* = 515).

Variable	Effect	95% CI	p
Age at enrollment (per 10‐year increase)	1.11	(0.47, 1.76)	< 0.001
Gender (male vs. female)	2.67	(0.90, 4.43)	0.0032
Race (Non‐White vs. White)	−1.57	(−3.98, 0.83)	0.2
Ethnicity (Hispanic/Latino vs. Non‐Hispanic/Non‐Latino)	1.66	(−3.20, 6.52)	0.5
Income (per 10 k increase)	0.83	(0.58, 1.08)	< 0.001
Education level (high school or less vs. college or higher)	0.02	(−1.94, 1.98)	0.98
Household members (per 1 person increase)	−0.70	(−1.5, 0.11)	0.089
Insurance			
Government vs. commercial	1.72	(−0.54, 3.98)	0.13
Self‐pay vs. commercial	−1.58	(−5.49, 2.3)	0.43
Interval from diagnosis to enrollment (per 12‐month increase)	0.15	(−0.12, 0.42)	0.27
Site of primary disease			
Extremity vs. trunk	−0.40	(−2.53, 1.72)	0.71
Head and neck vs. trunk	−0.81	(−4.34, 2.73)	0.65
Retroperitoneal vs. trunk	−0.53	(−3.10, 2.03)	0.68
Disease extent (metastatic vs. localized)	−2.89	(−4.85, −0.92)	0.0041
Recurrence/relapse (Yes vs. No)	−0.79	(−3.18, 1.60)	0.52

### 3.4 Association Between Psychosocial and Functional Impairment and Financial Toxicity

Among the 1125 participants who completed the COST measure, 832 completed all seven domains comprising the PROMIS‐57. Several demographic and clinical characteristics, as well as COST results, differed significantly between participants completing only the COST and those completing both the PROMIS‐57 and COST. Participants in the COST‐only group reported a lower median COST score (25.0 versus 28.0), had a smaller proportion of localized disease, and a lower rate of college or higher education. Marital status also differed significantly, with greater proportions of divorced and separated/widowed participants and a smaller proportion of never married participants in the COST‐only group (Table S1). Metastatic disease was less common among participants who completed only the COST (28.0%), compared to those who completed the COST and PROMIS‐57 (44.5%) (*p* = 0.037). Participants who completed the COST and PROMIS‐57 measures reported a higher level of education (College or Higher = 67.4%), compared to those who completed the COST alone (College or Higher = 60.6%) (*p* = 0.037).

Multivariable linear regression demonstrated that among the sub‐group of patients who completed the COST and PROMIS‐57, an increase in PROMIS‐57 Anxiety, Depression, Pain Interference, and Sleep Disturbance T‐scores (per 10‐point increase) was inversely associated with COST score, that is, higher FT by −0.77 (−1.45, −0.08; *p* = 0.028), −2.11 (−2.96, −1.26; *p* = < 0.001), −1.36 (−2.17, −0.55; *p* = 0.0011), and −0.68 (−1.35, −0.004; *p* = 0.049) points, respectively (Table 6).

**TABLE 6:** Multivariate linear regression model for COST scores among adult participants who completed all PROMIS domains (*n* = 832).

Variable	Effect	95% CI	*p*
Age at enrollment (per 10‐year increase)	0.81	(0.36, 1.26)	< 0.001
Gender (male vs. female)	0.57	(−0.62, 1.77)	0.35
Race (Non‐White vs. White)	−2.70	(−4.43, −0.97)	0.0023
Ethnicity (Hispanic/Latino vs. Non‐Hispanic/Non‐Latino)	−1.64	(−4.80, 1.52)	0.31
Income (per 10 k increase)	0.77	(0.62, 0.92)	< 0.001
Education level (high school or less vs. college or higher)	−0.15	(−1.49, 1.19)	0.83
Household members (per 1 person increase)	−0.22	(−0.76, 0.33)	0.43
Insurance			
Government vs. commercial	2.69	(1.13, 4.25)	< 0.001
Self‐pay vs. commercial	0.58	(−1.63, 2.79)	0.61
Treatment status (On vs. Off)	−1.38	(−2.78, 0.02)	0.054
Duration of time off treatment (per 12‐month increase)	−0.08	(−0.29, 0.13)	0.47
Site of primary disease			
Extremity vs. trunk	0.61	(−0.79, 2.01)	0.39
Head and Neck vs. trunk	0.92	(−1.43, 3.28)	0.44
Retroperitoneal vs. trunk	0.13	(−1.76, 2.01)	0.9
Disease extent (metastatic vs. localized)	−1.39	(−2.92, 0.13)	0.073
Recurrence/relapse (Yes vs. No)	0.47	(−1.03, 1.98)	0.54
PROMIS anxiety T‐score (per 10‐point increase)	−0.77	(−1.45, −0.08)	0.028
PROMIS depression T‐score (per 10‐point increase)	−2.11	(−2.96, −1.26)	< 0.001
PROMIS fatigue T‐score (per 10‐point increase)	−0.55	(−1.36, 0.26)	0.18
PROMIS pain interference T‐score (per 10‐point increase)	−1.36	(−2.17, −0.55)	0.0011
PROMIS physical function T‐score (per 10‐point increase)	0.004	(−0.839, 0.847)	0.99
PROMIS sleep disturbance T‐score (per 10‐point increase)	−0.68	(−1.35, −0.004)	0.049
PROMIS social roles T‐score (per 10‐point increase)	0.30	(−0.42, 1.01)	0.41

Among this sub‐group completing the COST and PROMIS 57, increasing age at enrollment (per 10‐year increase), increasing household income (per $10 000 increase), and government (versus commercial) sponsored health insurance was associated with increased COST scores by 0.81 (0.36, 1.26, *p* = < 0.001), 0.77 (0.62, 0.92, *p* = < 0.001), and 2.69 (1.13, 4.25, *p* = < 0.001) points, respectively, indicating lower FT. As was true in the whole cohort, non‐White race was associated with a decreased COST score by −2.70 (−4.43, −0.97, *p* = 0.0023) points, consistent with higher FT.

## 4 Discussion

In our cohort of 1125 sarcoma patients, FT was reported in close to half of participants, with 48.9% of participants on therapy (*n* = 515) and 41% of participants in off therapy surveillance (*n* = 610) reporting some level of FT. No prior US study has reported FT prevalence in sarcoma. Studies of other malignancies have reported a wide range of FT from 45% to 80% [38, 43, 44, 45, 46, 47].

Our study identified several demographic, disease, psychosocial, and functional factors that were associated with FT, including age, sex, race, household income, number of household members, metastatic disease, active treatment, and self‐reported anxiety, depression, pain interference, and sleep disturbance.

The association between age and FT has been evaluated among cohorts encompassing multiple cancer diagnoses, where similar to our study, younger age has consistently been associated with greater FT [18, 23, 37, 39, 40, 48, 49, 50, 51]. Sarcoma treatment is often prolonged, at times requiring hospitalization for therapy and treatment‐related complications, which can substantially disrupt employment, education, and opportunities for financial stability. Younger patients are particularly vulnerable to these burdens. They frequently face transitional points in their personal and professional lives that may limit access to income and insurance coverage [40]. Younger patients and survivors have fewer financial reserves, with limited time to accumulate savings or qualify for retirement benefits [52]. Education and work disruptions are notable among younger populations of cancer patients and survivors [40, 52, 53], which may contribute to FT. Additionally, with improving treatment and supportive care practices [3, 4, 5, 6], younger adults are living longer post‐treatment, resulting in greater cumulative financial demands over time.

There are limited data evaluating sex and its relationship to FT among sarcoma patients. One European study evaluated this association among patients with advanced soft tissue sarcoma and revealed that male participants experienced greater FT compared to females [29]. In contrast, our findings suggest that female patients experience greater FT. This observation is consistent with US‐based cohorts evaluating risk factors for FT among patients of diverse cancer types [39, 40, 54, 55]. Although not specific to sarcoma, women in the US generally face higher medical costs compared to men [56, 57, 58]. The added financial burden of a sarcoma diagnosis may therefore exacerbate this gap, contributing to greater FT.

As expected, our data demonstrate that increasing household income, across all groups, was associated with reduced FT. Similar findings were reported in cohorts involving common cancer types [23, 32, 35, 51, 59, 60, 61] as well as one large German study of sarcoma patients [27].

Few studies have evaluated the association between household size and FT among cancer patients [41, 62], none of which were performed among a US‐based cohort, nor exclusively in sarcoma patients. Our analysis demonstrates that among the overall cohort and participants off therapy, an increasing number of household members was an independent risk factor for greater FT, even when considering household income. This likely reflects that household income, a fixed resource, must stretch further with increasing numbers of members.

Data from our overall cohort reveal that non‐White race is associated with increased FT, compared to those who identify as White. This result is consistent with similar studies evaluating the influence of race on FT [21, 24, 39, 40, 43, 50, 60, 63, 64, 65]. We also tested the interaction term (race and treatment) in the model and found that the effect of race on FT did not differ between the “on therapy” and “off therapy” groups.

Disease extent (metastatic vs. localized) has rarely been examined as a risk factor for FT across cancer types [38, 66]. In our analysis, metastatic disease was significantly associated with lower COST scores (higher FT) among the overall cohort and participants receiving active therapy. This result is consistent with prior studies reporting higher financial burden among patients with other metastatic malignancies [67, 68, 69, 70, 71, 72]. For many cancer types, treatment for metastatic disease may involve higher intensity interventions with increasing frequency and morbidity of treatment, requiring greater supportive care and healthcare utilization [71, 73]. These factors likely contribute to greater costs and a reduced ability to work during treatment. Interestingly, data in our off‐therapy subgroup did not exhibit a similar significant association with FT and this may represent a combination of the effect of therapy diminishing over time and fewer patients and selection bias, where a lower proportion of patients with metastatic disease are long‐term survivors.

There are limited studies of psychosocial and functional correlates of FT. To date, no US‐based study has evaluated self‐reported anxiety, depression, pain interference, sleep disturbance, fatigue, physical function, or social activity with FT specifically among sarcoma patients. One study, conducted in the Netherlands, evaluated QoL and functioning in relation to FT among a cohort of patients treated with tyrosine kinase inhibitors for gastrointestinal stromal tumor (GIST). Notably, that study found that participants reporting symptoms of anxiety had higher odds of experiencing financial troubles, specifically paying expenses [28]. Among patients with other cancer types both during and after therapy, those experiencing financial hardship were more likely to report symptoms of anxiety and depression [74, 75, 76]. Greater fatigue and pain, as well as lower physical and social functioning, have also been shown to be associated with higher FT [77]. In one study of breast cancer patients, investigators also reported financial stress associated with difficulty falling asleep [78]. Our results among sarcoma patients, who have not been well represented in these studies, support an association between anxiety and depression, pain interference and sleep disturbance with FT, though the directionality of this association cannot be established by our study design. Prior studies suggest FT is likely a factor leading to functional/psychosocial distress; however, baseline QoL can also lead to subsequent financial hardship [75, 79]. This relationship may also sustain reinforcing feedback.

There are several limitations of this study. Despite the size of our cohort, there was considerable heterogeneity in sarcoma subtype and treatment, precluding disease subtype inclusion in the analysis. An additional limitation is that ours is a cross‐sectional study, limiting our ability to identify bidirectionality and reverse causation of resulting relationships. This study is a single center design, which decreases generalizability. Our study population is predominantly self‐described as White, limiting generalizability to diverse populations. Associations between race and outcomes reported here should therefore be interpreted with caution. Furthermore, participants who completed both the COST and PROMIS measures differed from noncompleters across several demographic characteristics, including race, education, marital status, and insurance status, suggesting a systematic bias toward socioeconomic advantage among responders (Table S2). This raises the possibility that financial toxicity burden may be underestimated in our cohort. It should be noted that our patient population reflects that reported by the State of Tennessee Cancer Registry, suggesting generalizability in this specific geographic region. Generalizability to populations with different healthcare systems, socioeconomic contexts, and greater racial and ethnic diversity remains uncertain and warrants further evaluation in larger, more diverse studies. Lastly, we were unable to include patient‐level objective financial metrics, such as out‐of‐pocket expenditures, medical debt, or employment disruption. These indicators have previously shown to be meaningful contributors to financial hardship among cancer patients and survivors [40, 80, 81, 82] and should be incorporated in future studies to provide a more comprehensive characterization of financial toxicity.

## 5 Conclusion

Our study represents the largest US‐based study of unselected patients with sarcoma across subtypes, both on and off therapy, with diverse demographic characteristics, including nearly 15% racial minorities, and supports the prevalence of and risk factors for FT identified in other cancer types.

We have identified several patient demographic and disease‐related risk factors associated with FT, among patients both receiving therapy and in post‐therapy surveillance. Recognizing individuals at greatest risk for FT can inform strategies to mitigate its contribution to cancer‐related adverse outcomes and patient QoL. Clinicians are often ill‐prepared to counsel patients on matters related to financial hardship. Likewise, patients are often reluctant to share their concerns [83, 84]. With identification of FT among patients, we may empower provider‐patient communication to identify financial barriers, expand access to patient and financial navigation services, increase awareness of psychosocial needs, and integrate FT screening into routine cancer care. Collectively, such efforts may have the potential to reduce FT and improve both patient well‐being and treatment outcomes. Future research should include characterization of specific contributors to FT among sarcoma patients, including item‐level analyses of the COST measure to identify which components of financial hardship are most prominent in this population, as well as qualitative assessments to capture the experiences of patients, survivors, and families that the COST may not fully reflect. Downstream, data from our study can inform the design of and lead to larger studies and interventions, which may potentially impact health policy in terms of out‐of‐pocket costs, at institutional, regional or national levels, such as has been performed for prescription medication pricing [20].

## Author Contributions

**Michael J. Robinson:** conceptualization, investigation, writing – original draft, methodology, writing – review and editing, formal analysis, project administration, supervision, resources. **Lili Sun:** formal analysis, writing – review and editing, methodology. **Tatsuki Koyama:** methodology, writing – review and editing, formal analysis, supervision. **Emma A. Schremp:** methodology, writing – review and editing, formal analysis, supervision, data curation, resources, project administration. **Grace Aronoff:** project administration, data curation, resources. **Tulio Guimaraes:** data curation, resources, project administration. **Taylor A. Stokes:** data curation, resources, project administration. **Elizabeth J. Davis:** writing – review and editing, data curation, resources. **Vicki Keedy:** data curation, resources, writing – review and editing. **Scott C. Borinstein:** writing – review and editing, resources, data curation. **Herbert S. Schwartz:** data curation, resources, writing – review and editing. **Jennifer L. Halpern:** writing – review and editing, resources, data curation. **Joshua M. Lawrenz:** writing – review and editing, data curation, resources. **Daniel Johnson:** data curation, resources. **Timothy Su:** resources, data curation, methodology, conceptualization. **Leo Luo:** resources, data curation. **Travis Osterman:** resources, data curation, methodology, conceptualization. **Tuya Pal:** supervision, funding acquisition, writing – review and editing, conceptualization. **Ben H. Park:** conceptualization, funding acquisition, writing – review and editing, supervision. **Xiao‐Ou Shu:** writing – review and editing, conceptualization, funding acquisition, supervision. **Debra L. Friedman:** conceptualization, funding acquisition, writing – review and editing, supervision. **Eric T. Shinohara:** conceptualization, funding acquisition, writing – review and editing, supervision, resources, project administration.

## Funding

This work was supported by the National Cancer Institute (4UH3CA260318‐03, P30CA06485, T32CA217834, 2T32CA160056, 5K12CA090625).

## Ethics Statement

The study followed ethical guidelines, obtained Institutional Review Board (IRB) Committee approval (#212419) at Vanderbilt University Medical Center, and got informed consent from human participants, adhering to principles like the Declaration of Helsinki.

## Conflicts of Interest

Michael J. Robinson, Lili Sun, Tatsuki Koyama, Emma A. Schremp, Grace Aronoff, Tulio Guimaraes, Taylor A. Stokes, Jennifer L. Halpern, Joshua M. Lawrenz, Daniel Johnson, Timothy Su, Leo Luo, Travis Osterman, Vicki Keedy, Herbert S. Schwartz, Tuya Pal, Xiao‐Ou Shu, Debra L. Friedman, and Eric T. Shinohara, declare no conflicts of interest. Elizabeth J. Davis reports consulting for Deciphera, AADI, and Cogent, and research funding from Cogent, Cornerstone, TopAlliance, Inhibrx, PharmaMar, NuMab, Servier, Mirati, Pheast, and Loxo. Scott C. Borinstein has served on an advisory board for Bayer and a data monitoring committee for Merck within the past 36 months. None of these relationships are associated with the work presented in this manuscript. Ben H. Park reports consulting fees and equity as a scientific advisory board member for Celcuity; consulting fees for Lilly, Astrazeneca, Astrin, Artera, and Caris; royalties from licensed products from Horizon Discovery; unpaid consulting for Tempus; and research contract for Natera. None of these relationships are associated with the work presented in this manuscript.

## Supporting information


**Table S1:** Comparison of participant demographic and disease among participants who completed the COST only versus the COST and PROMIS measures.
**Table S2:** Demographic and disease characteristics by completion of COST and PROMIS measures.

## Data Availability

The data that support the findings of this study are available from the corresponding author upon reasonable request.
