# Supplementary material for: Prevalence and Risk Factors for Financial Toxicity Among Sarcoma Patients During and Following Treatment Across the Lifespan
Source: Cancer Rep (Hoboken). 2026 Sep 30;9(10):e70686. doi: 10.1002/cnr2.70686 (PMC13627249; doi:10.1002/cnr2.70686)
Supplement: Supplementary file 1 [file CNR2-9-e70686-s001.docx]

| **Supplementary Table 1** Comparison of participant demographic and disease among participants who completed the COST only versus the COST and PROMIS measures | | | | | | |  |
| --- | --- | --- | --- | --- | --- | --- | --- |
| **Characteristic** | **COST Only (n=294)** | | **COST & PROMIS-57 (n = 831)** | | | **p-value** |  |
| **Median Age (IQR)** | 57.5 (43.6, 66.7) | | 55.9 (40.9, 67.3) | | | 0.31 |  |
| **Gender** |  |  |  |  | 0.41 | | |
| Male | 127 | 43.2% | 383 | 46.1% |  | | |
| Female | 167 | 56.8% | 448 | 53.9% |  |  |  |
| **Race** |  |  |  |  | 0.45 | | |
| White | 246 | 83.7% | 712 | 85.7% |  | | |
| Other | 48 | 16.3% | 119 | 14.3% |  |  |  |
| **Household Income** |  |  |  |  | 0.11 | | |
| < $25,000 | 32 | 10.9% | 71 | 8.5% |  | | |
| $25,000 ~ $45,000 | 45 | 15.3% | 114 | 13.7% |  | | |
| $45,000 ~ $65,000 | 32 | 10.9% | 121 | 14.6% |  | | |
| $65,000 ~ $70,000 | 18 | 6.1% | 44 | 5.3% |  | | |
| $70,000 ~ $90,000 | 38 | 12.9% | 89 | 10.7% |  | | |
| $90,000 ~ $120, 000 | 44 | 15.0% | 123 | 14.8% |  | | |
| $120,000 ~ $150,000 | 30 | 10.2% | 86 | 10.3% |  | | |
| > $150,000 | 31 | 10.5% | 143 | 17.2% |  | | |
| Missing | 24 | 8.2% | 40 | 4.8% |  | | |
| **Household Members** |  |  |  |  | 0.92 | | |
| 0 | 0 | 0.0% | 1 | 0.1% |  | | |
| 1 | 62 | 21.1% | 174 | 20.9% |  | | |
| 2 | 135 | 45.9% | 402 | 48.4% |  | | |
| 3 | 39 | 13.3% | 105 | 12.6% |  | | |
| 4 | 39 | 13.3% | 91 | 11.0% |  | | |
| 5 | 10 | 3.4% | 34 | 4.1% |  | | |
| 6 | 4 | 1.4% | 12 | 1.4% |  | | |
| 7 | 1 | 0.3% | 6 | 0.7% |  | | |
| 8 | 1 | 0.3% | 1 | 0.1% |  | | |
| Missing | 3 | 1.0% | 5 | 0.6% |  | | |
| **Disease Extent** |  |  |  |  | 0.037 | | |
| Localized | 211 | 71.8% | 649 | 78.1% |  | | |
| Metastatic | 82 | 27.9% | 181 | 21.8% |  | | |
| Missing | 1 | 0.3% | 1 | 0.1% |  | | |
| **Relapse/Recurrent Disease** |  |  |  |  | 0.67 | | |
| No | 231 | 78.6% | 662 | 79.7% |  | | |
| Yes | 62 | 21.1% | 165 | 19.9% |  | | |
| Missing | 1 | 0.3% | 4 | 0.5% |  | | |
| **Site of Primary Disease** |  |  |  |  | 0.66 | | |
| Trunk | 93 | 31.6% | 239 | 28.8% |  | | |
| Extremity | 144 | 49.0% | 405 | 48.7% |  | | |
| Head and Neck | 19 | 6.5% | 66 | 7.9% |  | | |
| Retroperitoneal | 37 | 12.6% | 119 | 14.3% |  | | |
| Missing | 1 | 0.3% | 2 | 0.2% |  | | |
| **Common Diagnoses** |  |  |  |  | 0.95 | | |
| Ewing Sarcoma | 11 | 3.7% | 34 | 4.1% |  | | |
| Desmoid | 28 | 9.5% | 63 | 7.6% |  | | |
| Gastrointestinal Stromal Tumor (GIST) | 28 | 9.5% | 79 | 9.5% |  | | |
| Osteosarcoma | 13 | 4.4% | 48 | 5.8% |  | | |
| Chondrosarcoma | 18 | 6.1% | 54 | 6.5% |  | | |
| Giant Cell Tumor | 9 | 3.1% | 19 | 2.3% |  | | |
| Synovial Sarcoma | 7 | 2.4% | 26 | 3.1% |  | | |
| Dedifferentiated Liposarcoma | 9 | 3.1% | 28 | 3.4% |  | | |
| Tenosynovial Giant Cell Tumor (TGCT) | 13 | 4.4% | 16 | 1.9% |  | | |
| Leiomyosarcoma | 30 | 10.2% | 70 | 8.4% |  | | |
| Myxofibrosarcoma | 20 | 6.8% | 43 | 5.2% |  | | |
| Myxoid Liposarcoma | 7 | 2.4% | 19 | 2.3% |  | | |
| Pleomorphic Sarcoma | 6 | 2.0% | 21 | 2.5% |  | | |
| Undifferentiated Pleomorphic Sarcoma | 16 | 5.4% | 55 | 6.6% |  | | |
| Well-Differentiated Liposarcoma | 9 | 3.1% | 46 | 5.5% |  | | |
| Other | 70 | 23.8% | 210 | 25.3% |  | | |
| **Marital Status** |  |  |  |  | 0.048 | | |
| Married | 203 | 69.0% | 581 | 69.9% |  | | |
| Divorced | 30 | 10.2% | 77 | 9.3% |  | | |
| Separated/Widowed | 28 | 9.5% | 47 | 5.7% |  | | |
| Never Married | 30 | 10.2% | 119 | 14.3% |  | | |
| Missing | 3 | 1.0% | 7 | 0.8% |  | | |
| **Employment status** |  |  |  |  | 0.71 | | |
| Employed | 133 | 45.2% | 408 | 49.1% |  | | |
| Unemployed | 37 | 12.6% | 99 | 11.9% |  | | |
| Retired | 105 | 35.7% | 291 | 35.0% |  | | |
| Missing | 19 | 6.5% | 33 | 4.0% |  | | |
| **Highest Level of Education** |  |  |  |  | 0.044 | | |
| College or Higher | 175 | 59.5% | 554 | 66.7% |  | | |
| High School or Less | 114 | 38.8% | 269 | 32.4% |  | | |
| Missing | 5 | 1.7% | 8 | 1.0% |  | | |
| **Insurance** |  |  |  |  | 0.48 | | |
| Commercial | 126 | 42.9% | 390 | 46.9% |  | | |
| Government | 134 | 45.6% | 350 | 42.1% |  | | |
| Self-Pay | 26 | 8.8% | 71 | 8.5% |  | | |
| Missing | 8 | 2.7% | 20 | 2.4% |  | | |
| **COST Score** |  |  |  |  | 0.006 | | |
| Median (Q1, Q3) | 25.0 (17.0, 32.0) | | 28.0 (18.0, 35.3) | |  | | |
| Range | 0.0 - 44.0 | | 0.0 - 44.0 | |  | | |

| **Supplementary Table 2** Demographic and disease characteristics by completion of COST and PROMIS measures | | | | | | |
| --- | --- | --- | --- | --- | --- | --- |
| **Characteristic** | **Did not complete both COST and PROMIS-57 (N=667)** | | **Completed both COST and PROMIS-57 (N=832)** | | **p-value** | |
| **Median Age (IQR)** | 56.7 (42.4, 67.5) | | 55.9 (40.9, 67.3) | | 0.39 | |
| **Gender** |  |  |  |  | 0.253 | |
| Male | 327 | 49.0% | 383 | 46.0% |  |  |
| Female | 340 | 51.0% | 449 | 54.0% |  |  |
| **Race** |  |  |  |  | 0.003 | |
| White | 532 | 79.8% | 713 | 85.7% |  |  |
| Other | 135 | 20.2% | 119 | 14.3% |  |  |
| **Household Income** |  |  |  |  |  |  |
| < $25,000 | 58 | 8.7% | 71 | 8.5% | 0.06 | |
| $25,000 ~ $45,000 | 80 | 12.0% | 114 | 13.7% |  |  |
| $45,000 ~ $65,000 | 61 | 9.1% | 121 | 14.5% |  |  |
| $65,000 ~ $70,000 | 34 | 5.1% | 44 | 5.3% |  |  |
| $70,000 ~ $90,000 | 52 | 7.8% | 89 | 10.7% |  |  |
| $90,000 ~ $120, 000 | 64 | 9.6% | 124 | 14.9% |  |  |
| $120,000 ~ $150,000 | 37 | 5.5% | 86 | 10.3% |  |  |
| > $150,000 | 64 | 9.6% | 143 | 17.2% |  |  |
| Missing | 217 | 32.5% | 40 | 4.8% |  |  |
| **Household Members** |  |  |  |  |  |  |
| 0 | 1 | 0.1% | 1 | 0.1% | 0.69 | |
| 1 | 121 | 18.1% | 174 | 20.9% |  |  |
| 2 | 223 | 33.4% | 403 | 48.4% |  |  |
| 3 | 69 | 10.3% | 105 | 12.6% |  |  |
| 4 | 57 | 8.5% | 91 | 10.9% |  |  |
| 5 | 18 | 2.7% | 34 | 4.1% |  |  |
| 6 | 5 | 0.7% | 12 | 1.4% |  |  |
| 7 | 1 | 0.1% | 6 | 0.7% |  |  |
| 8 | 1 | 0.1% | 1 | 0.1% |  |  |
| Missing | 171 | 25.6% | 5 | 0.6% |  |  |
| **Disease Extent** |  |  |  |  | 0.08 | |
| Localized | 490 | 73.5% | 649 | 78.0% |  |  |
| Metastatic | 171 | 25.6% | 181 | 21.8% |  |  |
| Missing | 6 | 0.9% | 2 | 0.2% |  |  |
| **Relapse/Recurrent Disease** |  |  |  |  | 0.79 | |
| No | 535 | 80.2% | 662 | 79.6% |  |  |
| Yes | 128 | 19.2% | 165 | 19.8% |  |  |
| Missing | 4 | 0.6% | 5 | 0.6% |  |  |
| **Site of Primary Disease** |  |  |  |  | 0.62 | |
| Trunk | 191 | 28.6% | 239 | 28.7% |  |  |
| Extremity | 333 | 49.9% | 405 | 48.7% |  |  |
| Head and Neck | 41 | 6.1% | 66 | 7.9% |  |  |
| Retroperitoneal | 97 | 14.5% | 119 | 14.3% |  |  |
| Missing | 5 | 0.7% | 3 | 0.4% |  |  |
| **Common Diagnoses** |  |  |  |  | 0.70 | |
| Ewing Sarcoma | 28 | 4.2% | 34 | 4.1% |  |  |
| Desmoid | 61 | 9.1% | 63 | 7.6% |  |  |
| Gastrointestinal Stromal Tumor (GIST) | 51 | 7.6% | 79 | 9.5% |  |  |
| Osteosarcoma | 27 | 4.0% | 48 | 5.8% |  |  |
| Chondrosarcoma | 44 | 6.6% | 54 | 6.5% |  |  |
| Giant Cell Tumor | 0 | 0.0% | 1 | 0.1% |  |  |
| Synovial Sarcoma | 20 | 3.0% | 26 | 3.1% |  |  |
| Dedifferentiated Liposarcoma | 26 | 3.9% | 28 | 3.4% |  |  |
| Tenosynovial Giant Cell Tumor (TGCT) | 21 | 3.1% | 16 | 1.9% |  |  |
| Leiomyosarcoma | 60 | 9.0% | 70 | 8.4% |  |  |
| Myxofibrosarcoma | 37 | 5.5% | 43 | 5.2% |  |  |
| Myxoid Liposarcoma | 13 | 1.9% | 19 | 2.3% |  |  |
| Pleomorphic Sarcoma | 15 | 2.2% | 21 | 2.5% |  |  |
| Undifferentiated Pleomorphic Sarcoma | 32 | 4.8% | 55 | 6.6% |  |  |
| Well-Differentiated Liposarcoma | 22 | 3.3% | 46 | 5.5% |  |  |
| Other | 209 | 31.3% | 228 | 27.4% |  |  |
| Missing | 1 | 0.1% | 1 | 0.1% |  |  |
| **Marital Status** |  |  |  |  | 0.076 | |
| Married | 336 | 50.4% | 582 | 70.0% |  |  |
| Divorced | 55 | 8.2% | 77 | 9.3% |  |  |
| Separated/Widowed | 45 | 6.7% | 47 | 5.6% |  |  |
| Never Married | 63 | 9.4% | 119 | 14.3% |  |  |
| Missing | 168 | 25.2% | 7 | 0.8% |  |  |
| **Employment status** |  |  |  |  |  |  |
| Employed | 238 | 35.7% | 409 | 49.2% | 0.899 | |
| Unemployed | 62 | 9.3% | 99 | 11.9% |  |  |
| Retired | 177 | 26.5% | 291 | 35.0% |  |  |
| Missing | 190 | 28.5% | 33 | 4.0% |  |  |
| **Highest Level of Education** |  |  |  |  | 0.001 | |
| College or Higher | 271 | 40.6% | 555 | 66.7% |  |  |
| High School or Less | 226 | 33.9% | 269 | 32.3% |  |  |
| Missing | 170 | 25.5% | 8 | 1.0% |  |  |
| **Insurance** |  |  |  |  |  |  |
| Commercial | 266 | 39.9% | 390 | 46.9% | 0.049 | |
| Government | 311 | 46.6% | 350 | 42.1% |  |  |
| Self-Pay | 57 | 8.5% | 71 | 8.5% |  |  |
| Missing | 33 | 4.9% | 21 | 2.5% |  |  |
